# Canal of Nuck hernia containing the internal reproductive organs of both gender in a neonate with disorder of sex development: a rare case presentation

**DOI:** 10.1259/bjrcr.20230003

**Published:** 2023-04-19

**Authors:** Kamal H. Attia, Mehad H. Felemban, Ahmad A. Al Boukai, Latifah A. Alfahad

**Affiliations:** 1 Department of Radiology and Medical Imaging, King Saud University Medical City, Riyadh, Saudi Arabia

## Abstract

The combination of disorder of sex development and canal of Nuck hernia, in which the hernial sac contains the internal reproductive organs (gonads) of both genders, is exceedingly rare. We present a unique case of a neonate who presented with ambiguous genitalia and a lump in the left inguinal region. The child underwent various radiographic procedures and hernial repair. Blood work-up, karyotyping, and histopathological analysis from gonads confirmed the diagnosis of Ovotesticular disorder of sex development. Subsequently, the child had a reconstructive vaginoplasty, and the final decision regarding gender assignment will be made after assessing mental and sexual behavior in early childhood.

## Case presentation

A 2-month-old baby was brought to our hospital by the parents concerned about the shape of their child’s external genitalia and a lump in the left inguinal region. On physical examination, the child had abnormal external genitalia and labioscrotal folds. A large chordae phallus measured 2.5 cm with an atretic urethral opening at its tip and a small urogenital opening in the perineum. There was a focal non-tender soft lump in the left inguinal region extending toward the left labioscrotal fold. The lump was not reducible, and the overlying skin was normal—no palpable testicles. The family history was negative for a similar condition. The pregnancy and delivery were uneventful. The laboratory tests revealed a high level of 17-hydroxyprogesterone, more than 16 nmol/mL [the normal range in our institution is 0.35–4.13 nmol/L]. The ACTH stimulation test was normal, with a good cortisol response (30 min: 766 nmol/L, 60 min: 1070 nmol/L) [normal: more than 500 nmol/L]. Based on the previous laboratory results, congenital adrenal hyperplasia (CAH), the most common cause of DSD, was excluded. Furthermore, the patient had an increased level of testosterone following the HCG stimulation test (baseline: 6.67 nmol/L, after stimulation: 20.50 nmol/L), indicating the presence of functioning testicular tissue.

## Investigations

**Figure 1.**
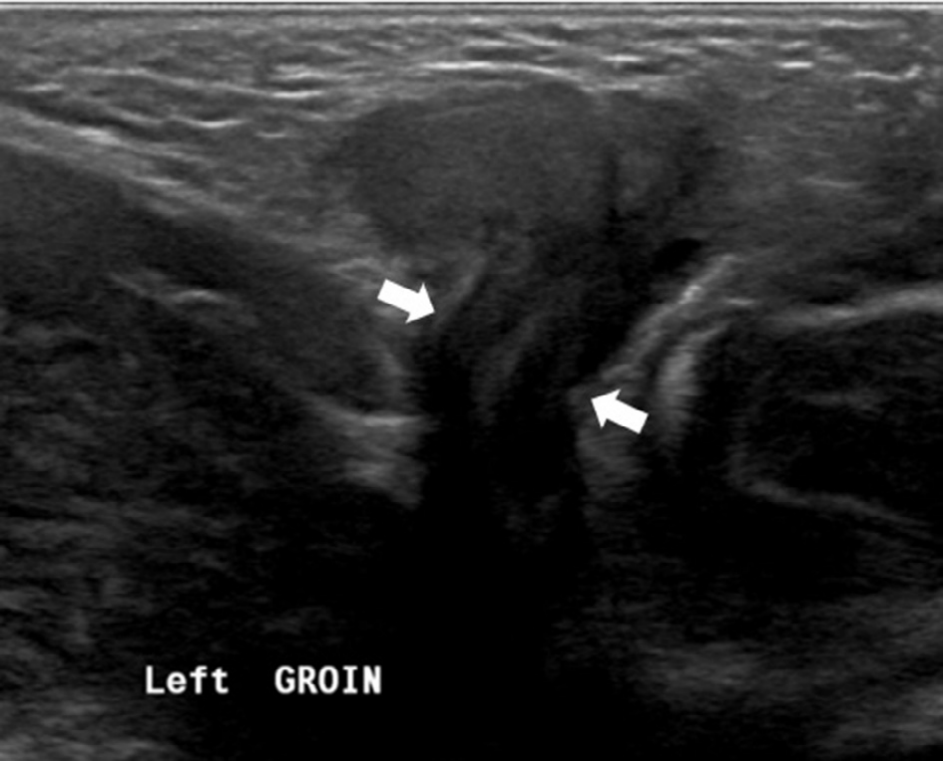
Grayscale ultrasound image showed a defect in the left inguinal area with a herniated uterus.

**Figure 2.**
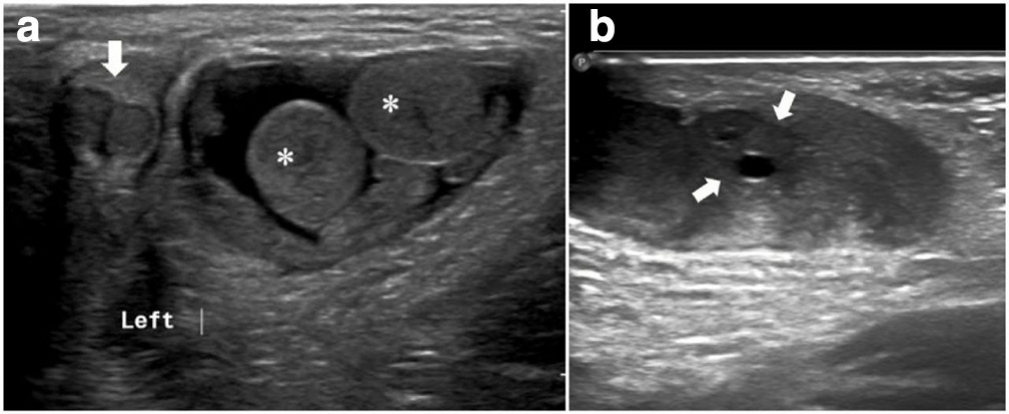
(a) Grayscale ultrasound images. A hernial sac containing two testicles (asterisk) and a partially scanned phallus with two corpora cavernosum (arrow). (b) An ovary containing small peripheral follicles (arrows).

Ultrasound evaluation showed a defect in the left inguinal region and a hernial sac extending downwards toward the labioscrotal fold. An elongated structure passes through the defect toward the bladder neck, with a central echogenic strip representing a herniated uterus (Figure 1). Three moderately echogenic structures were identified. Two are oval-shaped with thin echogenic capsules resembling testes, and a smaller one contains small follicles representing an ovary (Figure 2 a and b). No intra-abdominal gonads. Kidneys and adrenal glands were normal.

**Figure 3.**
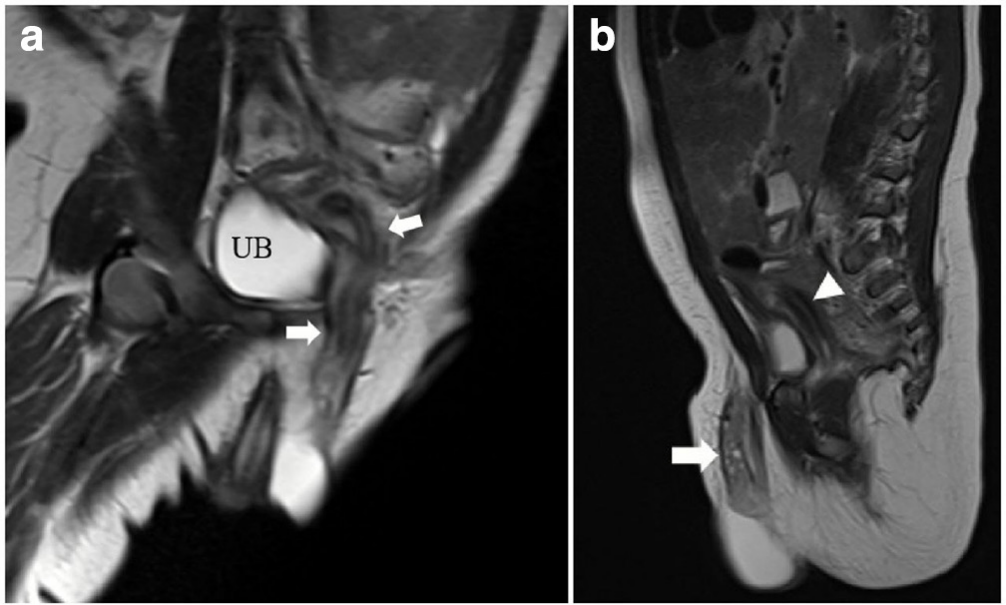
High-resolution *T*_2_ weighted MRI. (a) Coronal oblique image showing an elongated herniated uterus with normal zonal anatomy. (b) Sagittal image showing an ovary with multiple follicles (arrows) within the hernial sac and part of the uterus (arrowheads) posterior to the urinary bladder. UB, urinary bladder.

**Figure 4.**
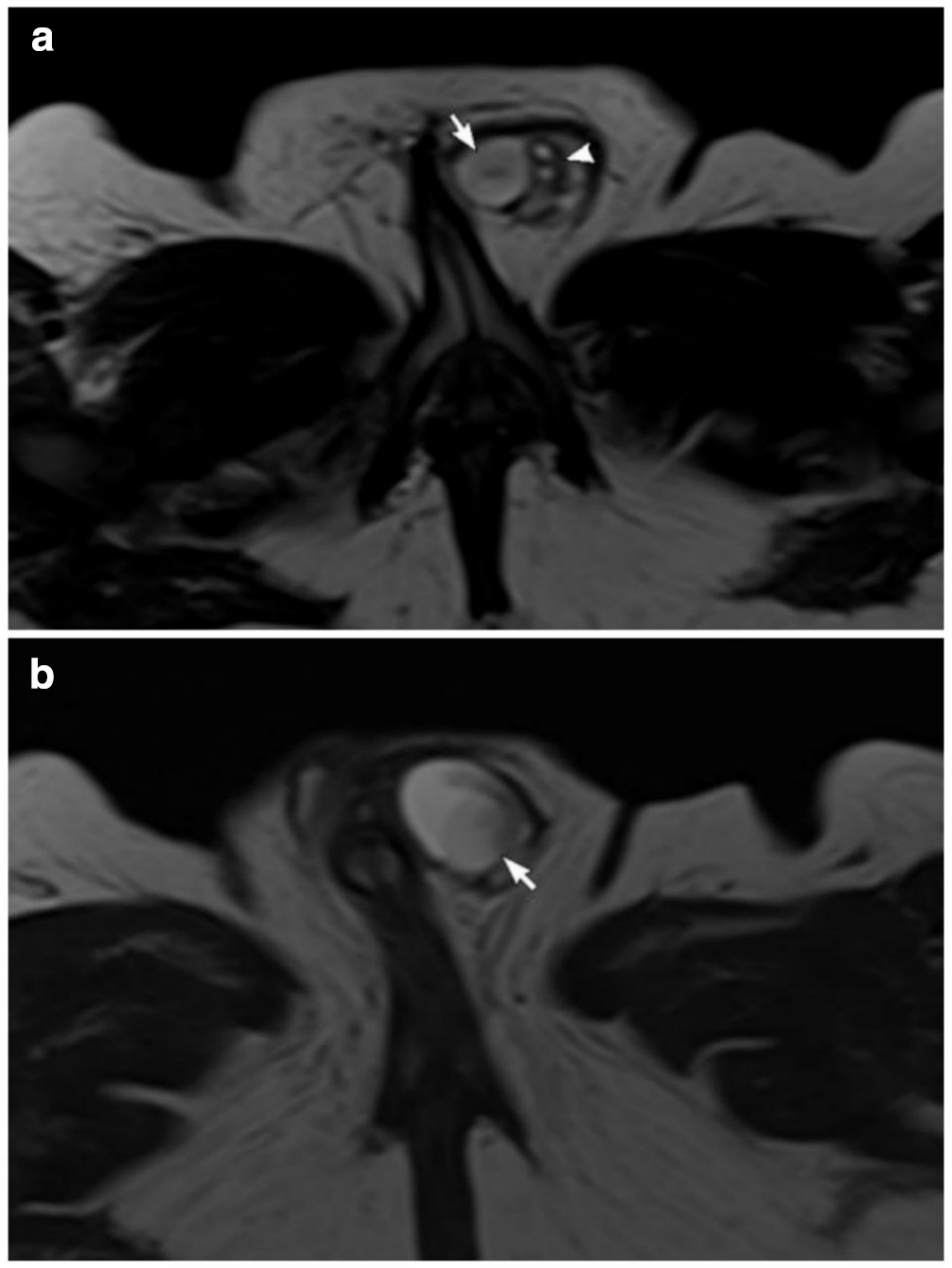
High-resolution *T*_2_ weighted MRI (a, b). Axial cuts in two different levels showing the testicles(arrows). Two small extratesticular cysts lateral to the testis (arrowhead) that could represent follicles in a small ovary or epididymal cysts.

A high-resolution pelvic MRI confirmed the presence of an elongated herniated uterus with normal zonal anatomy, an ovary (Figure 3a and b), and two testicles within the hernial sac (Figure 4a and b) . Vaginal and urethral openings could not be identified clearly. No associated vertebral or spinal cord anomalies.

**Figure 5.**
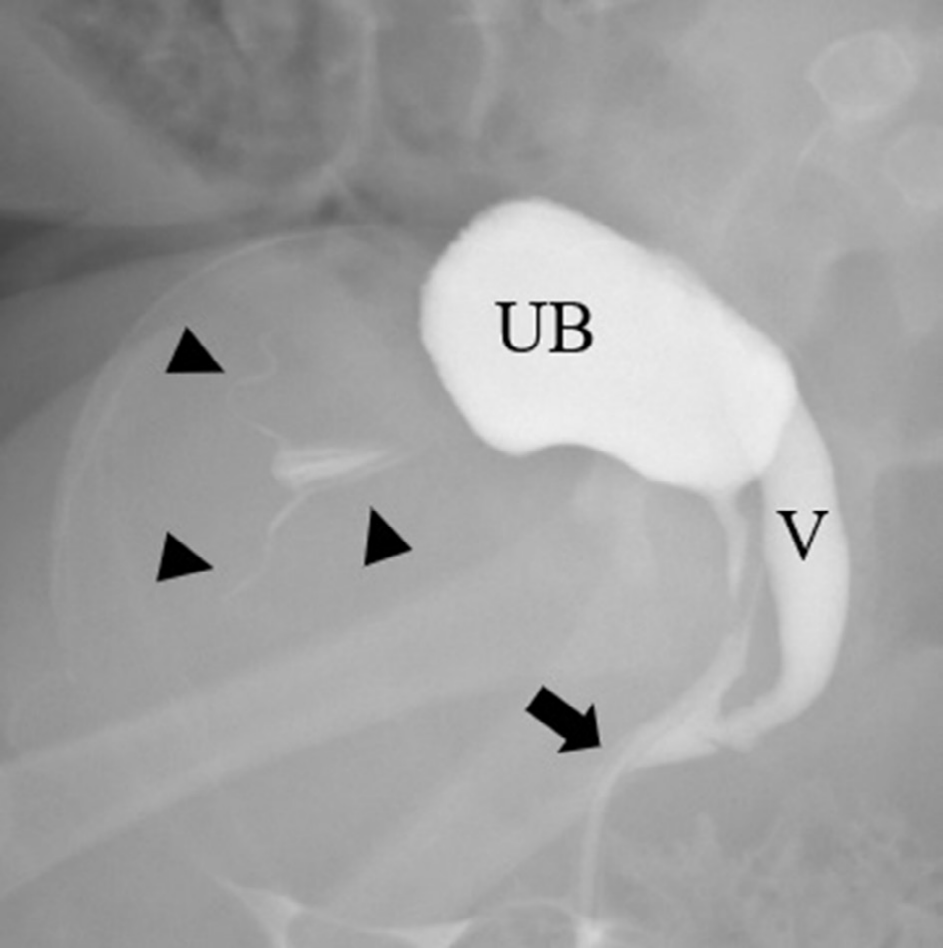
A selected image from fluoroscopic genitogram showing a urogenital sinus with a short common channel (arrow) and opacification of the urinary bladder (UB), vagina (V), endometrial cavity, and fallopian tubes (arrowheads).

A fluoroscopic genitogram through the single urogenital opening in the perineum showed a urogenital sinus with a short common channel. The endometrial cavity and fallopian tubes were opacified by contrast within the hernial sac (Figure 5).

The surgeon confirmed the presence of a second ovary within the hernial sac during diagnostic laparoscopy in addition to the previous contents mentioned by imaging. Karyotype testing revealed 46 XX, and tissue biopsy from gonads was consistent with a separated form of bilobated ovotestis.

## Differential diagnosis

The differential diagnosis for an inguinal lump is broad and includes inguinal hernia, hydrocele, lymphadenopathy, infection/abscess, inguinal gonad, and benign or malignant neoplasms.

## Diagnosis

Canal of Nuck hernia containing the internal reproductive organs in a child with Ovotesticular-Disorders of sex development (OT-DSD) based on karyotype, laboratory, and histopathology results.

## Treatment and outcome

The multidisciplinary team discussed the case thoroughly and explained it in detail to the family, including possible future consequences. The parents and the team decided to raise the baby as a female. Therefore, the child had reconstructive vaginoplasty. An elective orchiectomy will be performed at the pre-pubertal age after assessing the child’s mental and sexual behavior.

## Discussion

Disorders of sex development (DSD) refer to a group of diseases characterized by different pathophysiological changes resulting in abnormal development of gonads, external genital, or both with variant clinical manifestation.^1,2^ The patient usually develops secondary sexual characteristics incompatible with the assigned gender.The most frequent causes of disorders of sex development in individuals with the 46XX karyotype includes congenital adrenal hyperplasia, maternal androgens, and ovotesticular diseases.^3^

Ovotesticular DSD (OT-DSD) is one of the rarest DSDs in which both internal reproductive organs are present, either separated (ovaries and testes) or integrated (ovotestes).^4^ Patients with 46 XX karyotype have a broad spectrum of clinical presentations making it difficult to diagnose based on clinical examination and phenotype alone.^5^ The diagnostic approach starts with background history, clinical examination, laboratory testing, genetic analysis, and radiological evaluation. If no precise diagnosis is reached using non-invasive methods, surgical intervention and gonadal biopsy are performed.^5^

The typical physical manifestation of DSD is ambiguous genitalia. If a penile phallus is present, the urethral opening is usually abnormal in location (hypospadias). Testicles, if present, are more likely to be undescended. While a clear vaginal opening is not always visible by local inspection, it is usually evident on ultrasound, and a hypoplastic uterus is present.^4,5^

Radiology has a critical role in assessing the internal genitalia of DSDs patient. Ultrasound is the first modality of choice in most cases. It is non-invasive, easily accessible, and readily available in most hospitals. It carries no radiation risk or exposure to contrast material. It helps identify the gonads and the presence or absence of the uterus. The adrenal glands and kidneys are also evaluated by ultrasound. The main disadvantages are being operator-dependent and limited intra-abdominal assessment in some cases. High-resolution pelvic MRI has superior soft tissue resolution and multiplanar imaging capabilities. It can clarify the internal abdominal anatomy, localize non-palpable ectopic gonads or streak gonads, and detect the presence or absence of intra-abdominal reproductive organs like the uterus and prostate. Fluoroscopic Genitogram and voiding cystourethrogram have a role in complex cases. They are beneficial in delineating the urogenital sinus if associated with DSD. They are also helpful in identifying male or female urethra, the length of the common channel, and the level at which the vagina opens into the sinus.^5,6^

The Canal of Nuck hernia represents the continuation of an outpouching parietal peritoneum through the inguinal canal to labia majora. Usually, the hernia contains a bowel loop or peritoneal fat.^7^ Sometimes, the hernial sac includes the uterus, fallopian tubes, or ovaries. Herniation of ovaries carries an increased risk of complications such as incarceration and torsion; therefore, urgent surgical intervention is required for such cases.^8,9^

## Learning points

- DSD is a complex subject, and radiology is essential in diagnosing.
- The investigation and management of DSD should be tailored case-by-case and requires a multidisciplinary approach.
- Canal of Nuck hernia is uncommon and should be considered in young females with inguinal/labial swelling.
- Herniation of internal reproductive organs into the canal of Nuck hernia is rare.
